# Supplementary material for: 2D Monomolecular Nanosheets Based on Thiacalixarene Derivatives: Synthesis, Solid State Self-Assembly and Crystal Polymorphism
Source: Nanomaterials (Basel). 2020 Dec 14;10(12):2505. doi: 10.3390/nano10122505 (PMC7764881; doi:10.3390/nano10122505)
Supplement: Supplementary file 1 [file nanomaterials-10-02505-s001.pdf]

# Supporting Information

# for

# 2D monomolecular nanosheets based on thiacalixarene derivatives: synthesis, solid state self-assembly and crystal polymorphism

Alena A. Vavilova, Pavel L. Padnya, Timur A. Mukhametzyanov, Aleksey V. Buzyurov, Konstantin S. Usachev, Daut R. Islamov, Marat A. Ziganshin, Artur E. Boldyrev and Ivan I. Stoikov*

Address: Kazan Federal University, A.M. Butlerov Chemical Institute, 420008 Kremlevskaya, 18, Kazan, Russian Federation

Email: Stoikov Ivan Ivanovich - Ivan.Stoikov@mail.ru

* Corresponding author

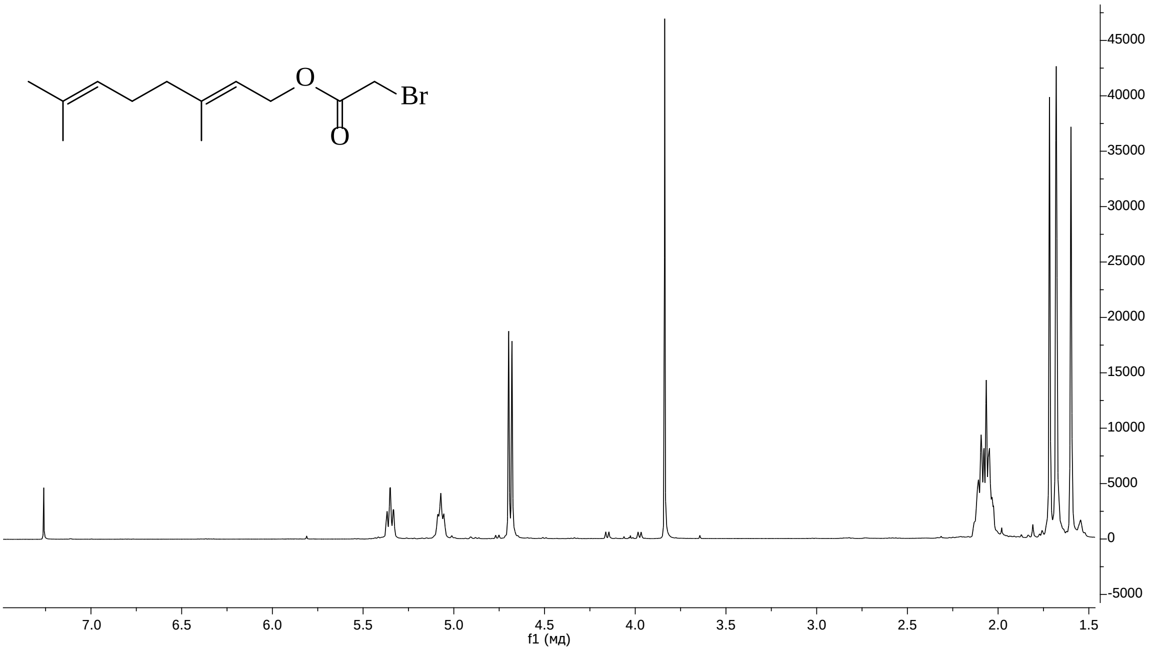


**Figure S1.** $^{1}$H NMR spectrum of geranyl bromoacetate **2** ($CDCl_3$, 298 K, 400 MHz).

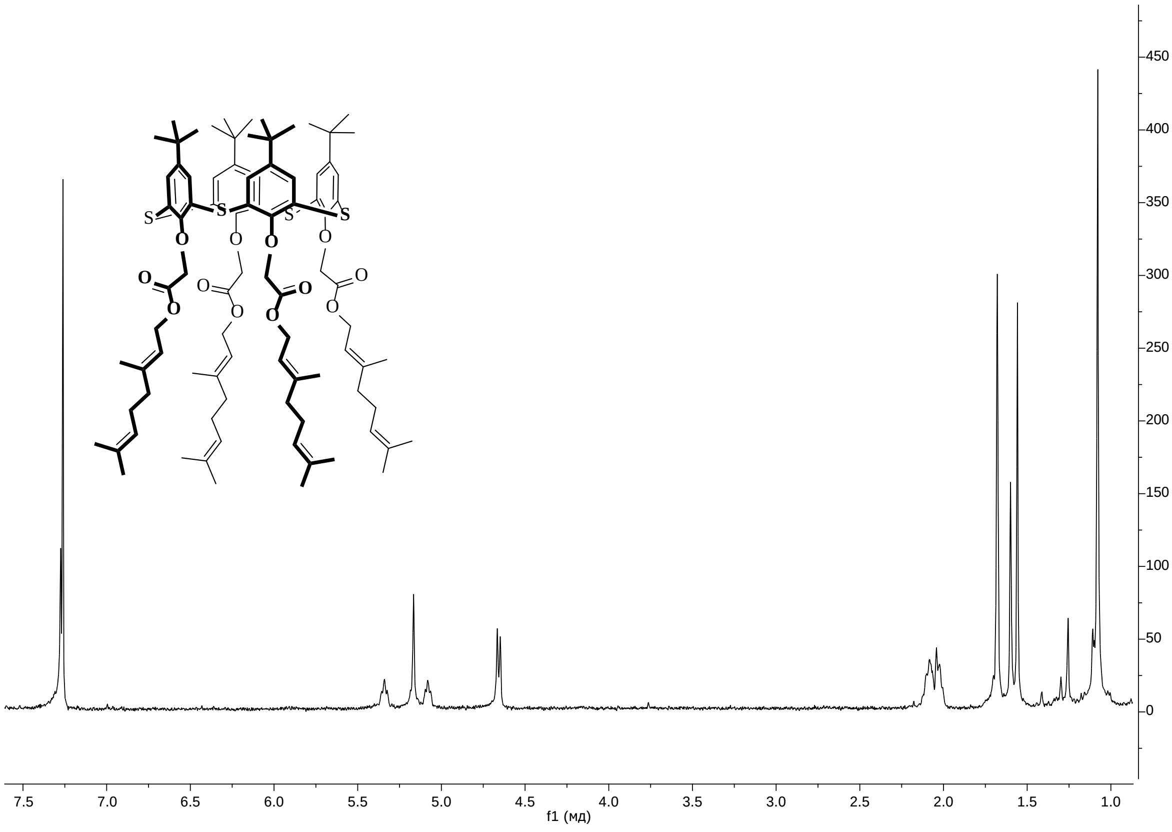


**Figure S2.** $^{1}$H NMR spectrum of thiacalix[4]arene **4** in *cone* conformation ($CDCl_3$, 298 K, 400 MHz).

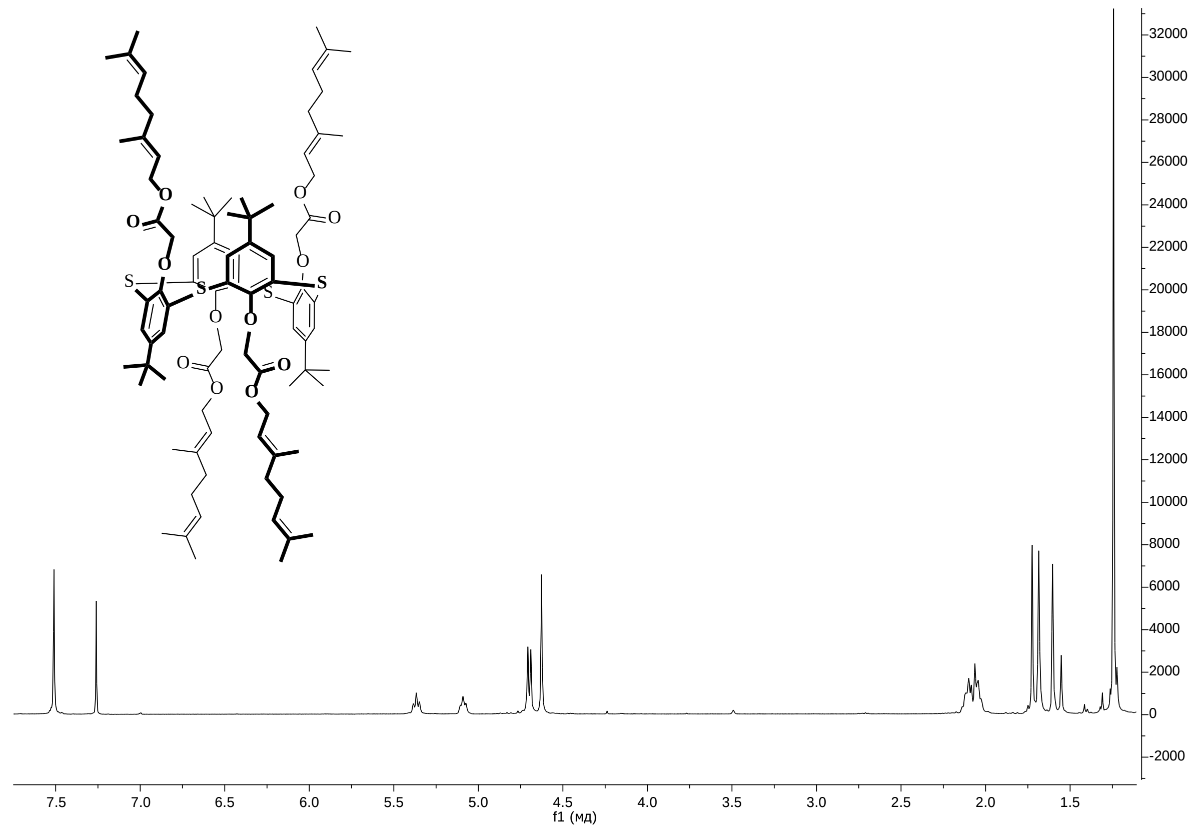


**Figure S3.** $^{1}$H NMR spectrum of thiacalix[4]arene **5** in *1,3-alternate* conformation ($CDCl_3$, 298 K, 400 MHz).

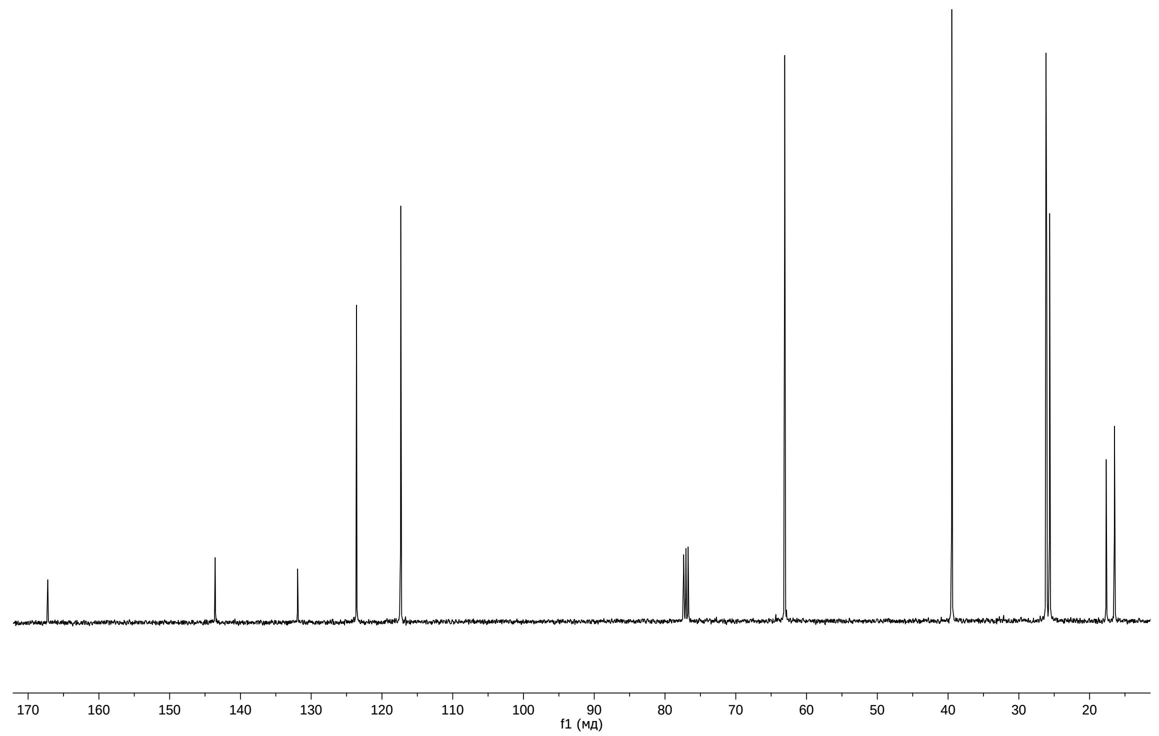


**Figure S4.** $^{13}$C NMR spectrum of geranyl bromoacetate **2** ($CDCl_3$, 298 K, 100 MHz).

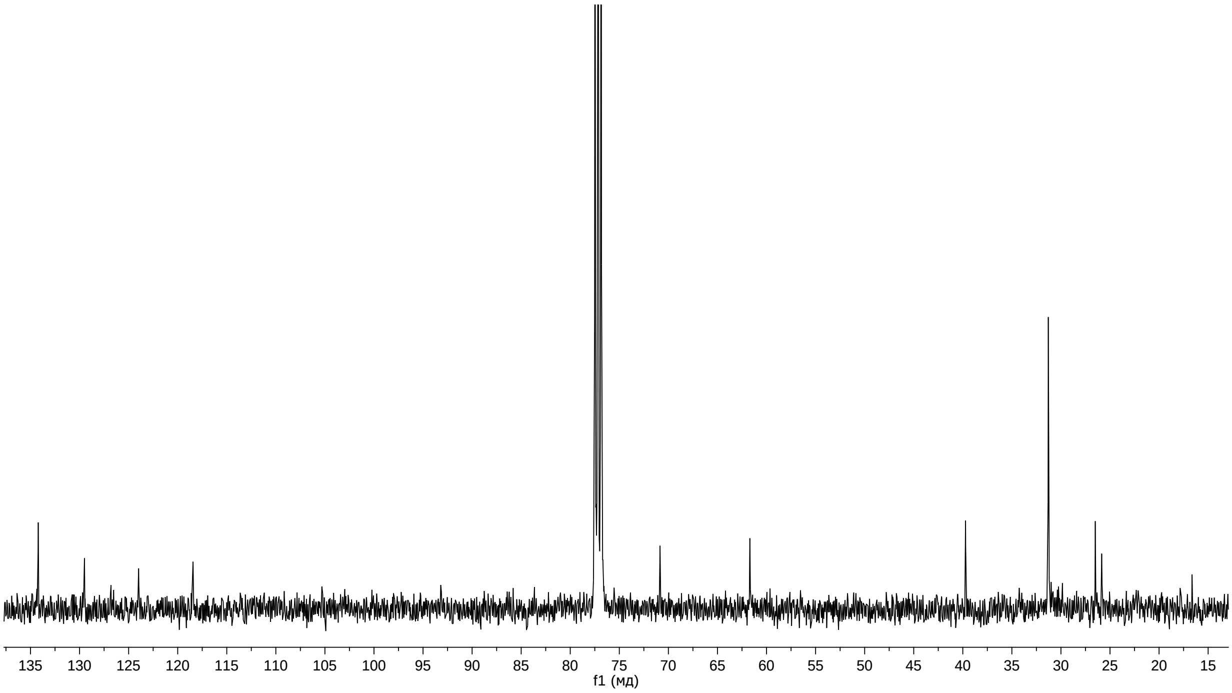


**Figure S5.** $^{13}C$ NMR spectrum of thiacalix[4]arene **4** in *cone* conformation ($CDCl_3$, 298 K, 100 MHz).

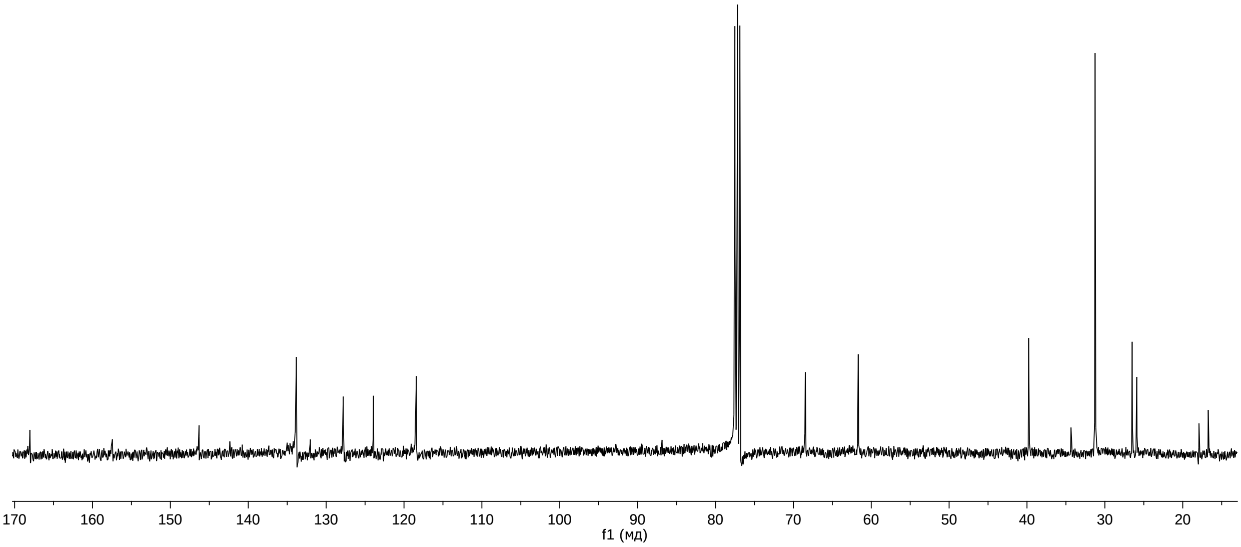


**Figure S6.** $^{13}C$ NMR spectrum of thiacalix[4]arene **5** in *1,3-alternate* conformation ($CDCl_3$, 298 K, 100 MHz).

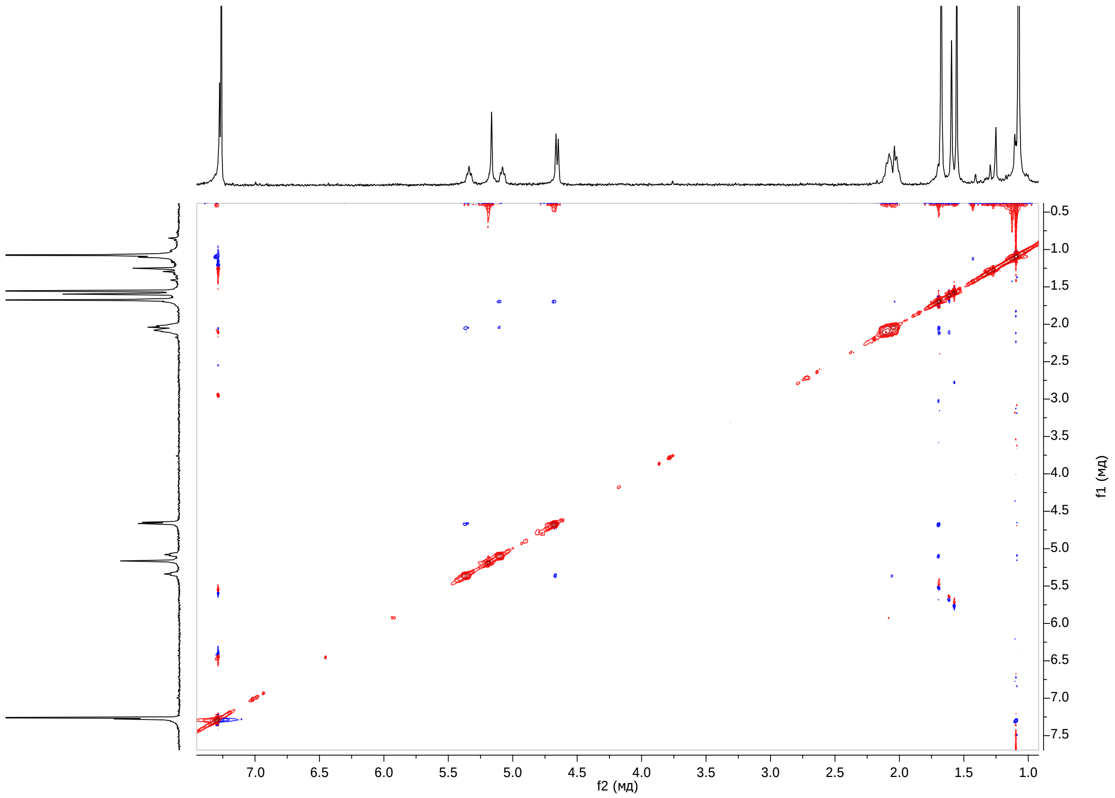


**Figure S7.** 2D NMR NOESY $^{1}H$-$^{1}H$ spectrum of thiacalix[4]arene **4** in *cone* conformation ($CDCl_3$, 298 K, 400 MHz).

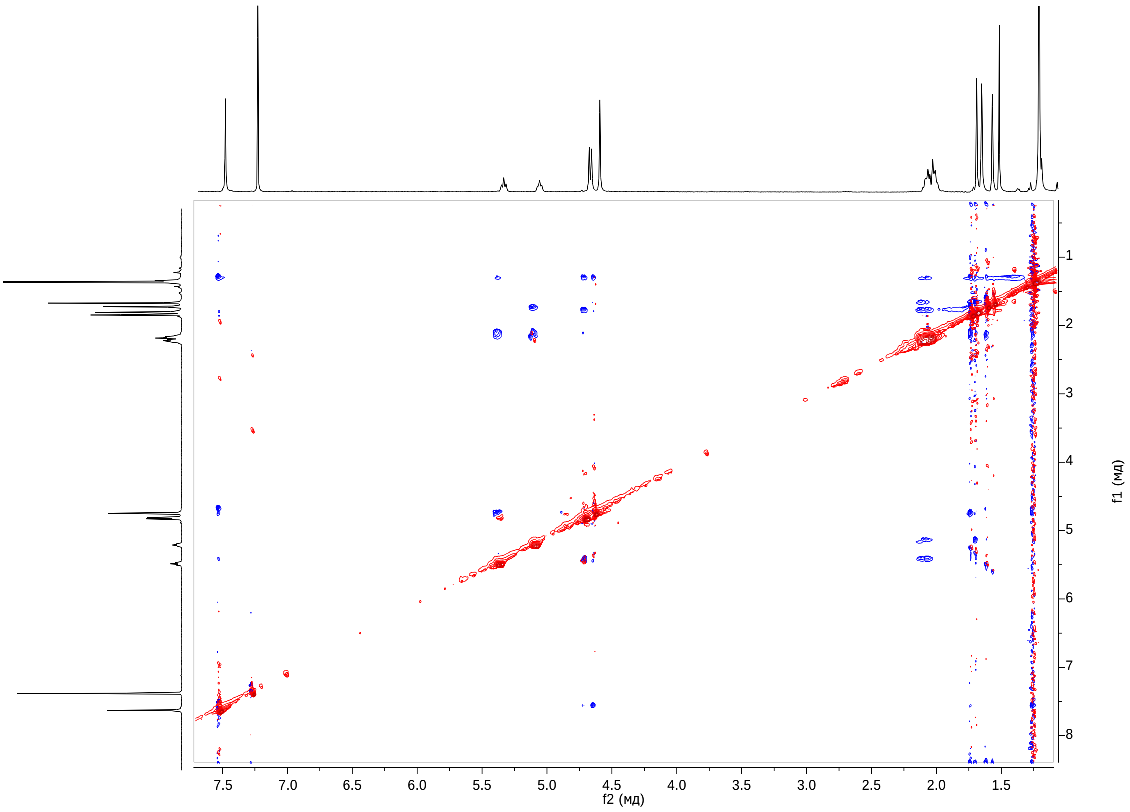


**Figure S8.** 2D NMR NOESY $^{1}H$-$^{1}H$ spectrum of thiacalix[4]arene **5** in *1,3-alternate* conformation ($CDCl_3$, 298 K, 400 MHz).

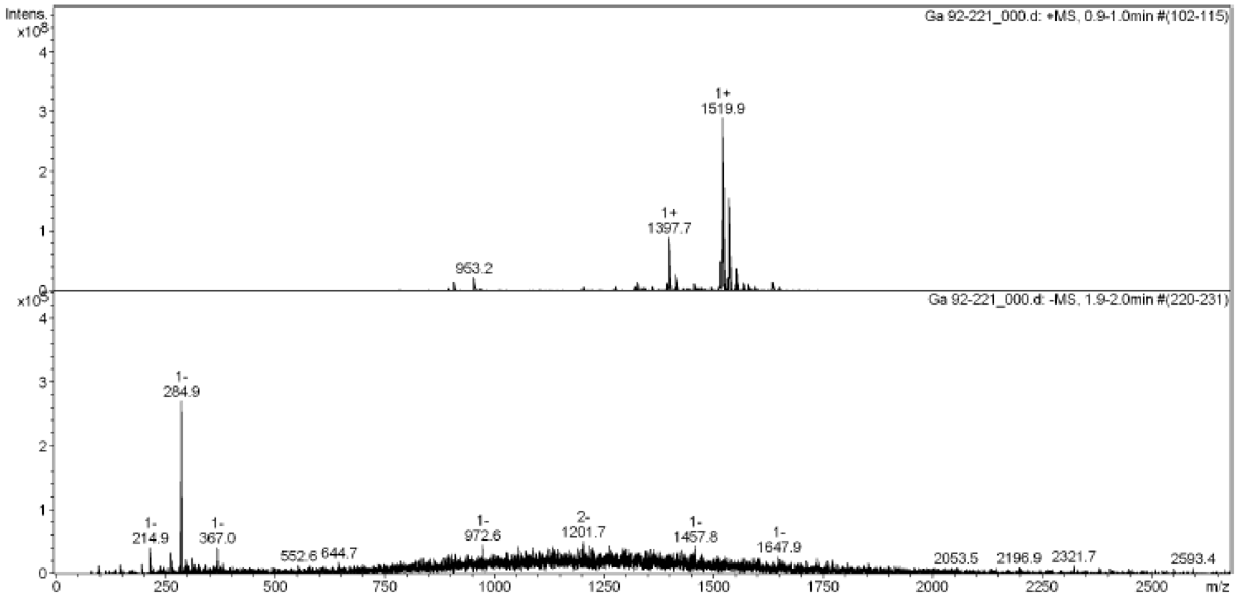


**Figure S9.** Mass spectrum ESI of thiacalix[4]arene **4** in *cone* conformation.

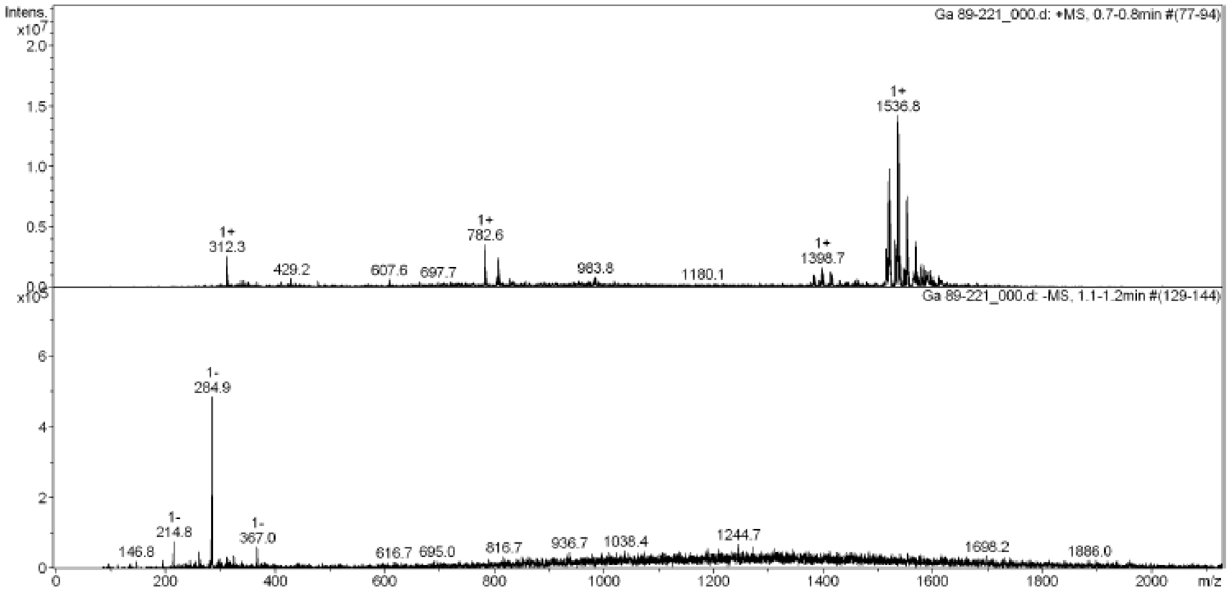


**Figure S10.** Mass spectrum ESI of thiacalix[4]arene **5** in *1,3-alternate* conformation.

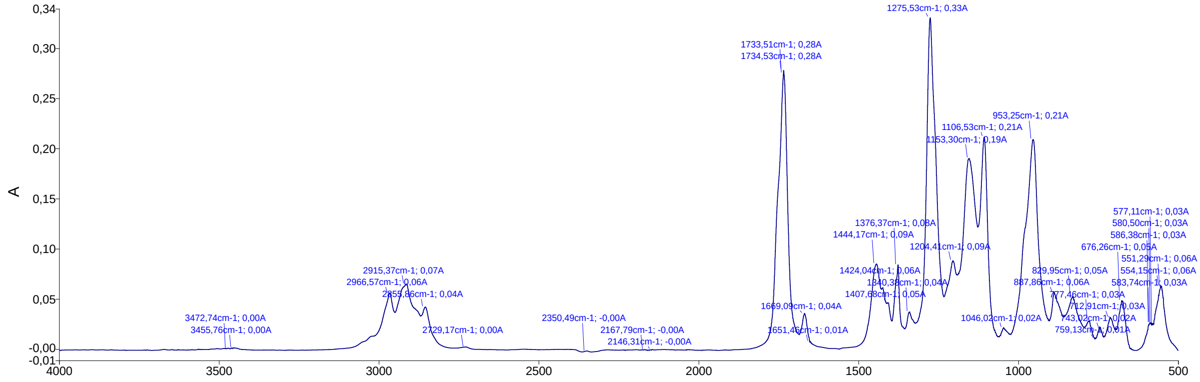


**Figure S11.** ATR FTIR spectrum of geranyl bromoacetate **2**.

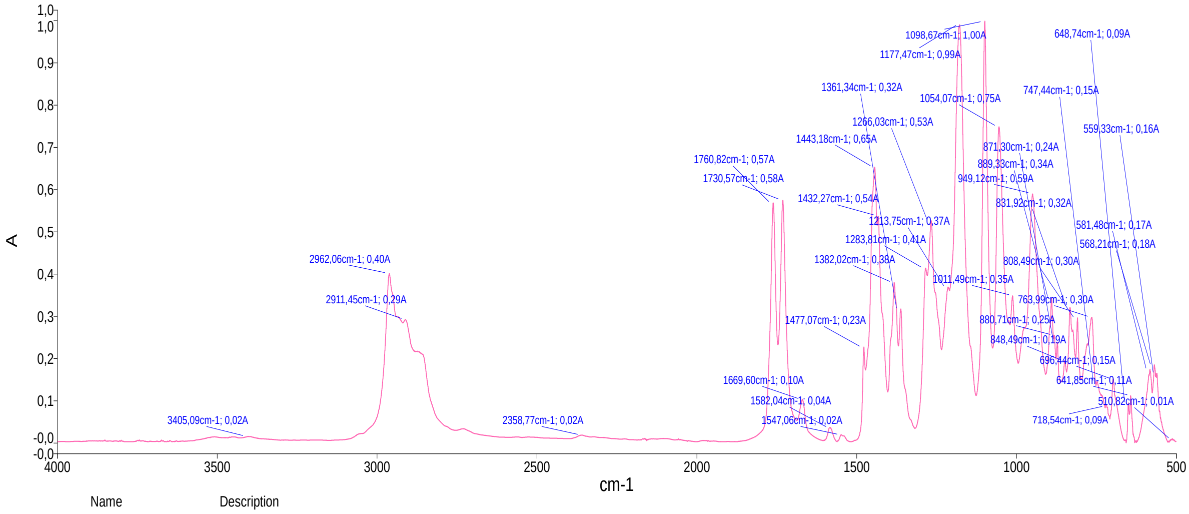


**Figure S12.** ATR FTIR spectrum of thiacalix[4]arene **4** in *cone* conformation.

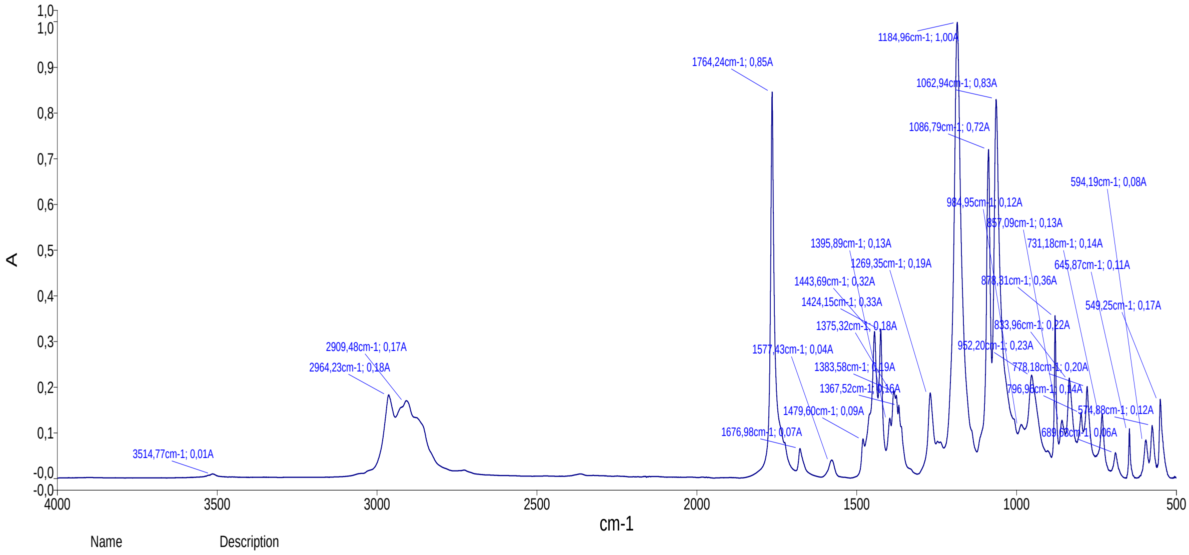


**Figure S13.** ATR FTIR spectrum of thiacalix[4]arene **5** in *1,3-alternate* conformation.

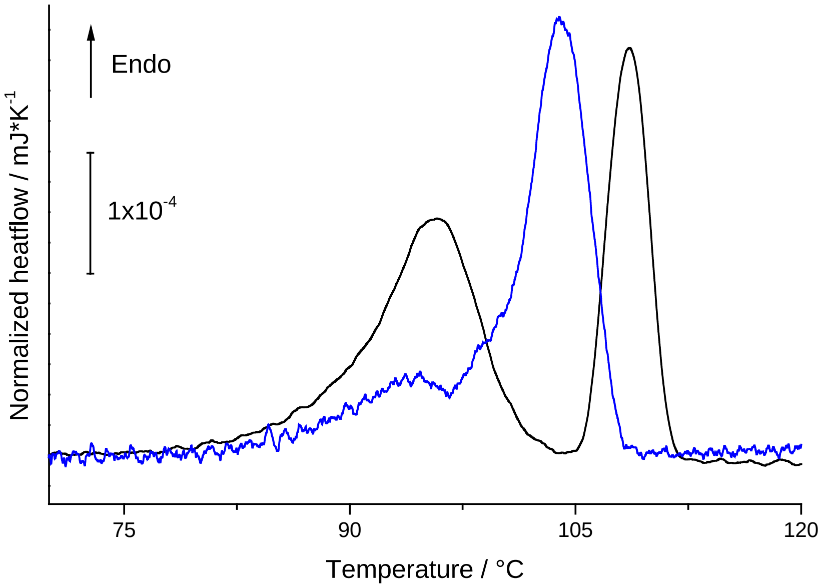


**Figure S14.** Data of FSC analysis: Heating scans of different samples of compound **5** crystallized on the chip-sensor. 100 K/s heating rate.

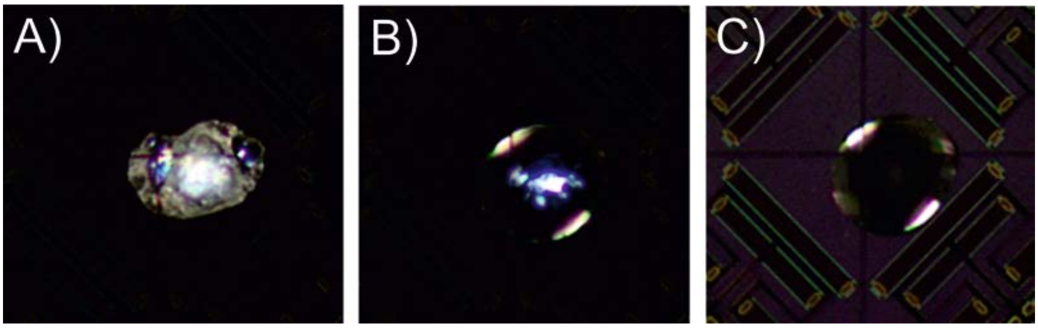


**Figure S15.** The images of microcrystalline sample **4** (*cone*) under polarized light at heating to 53°C (A), 60°C (B) and 63°C (C).

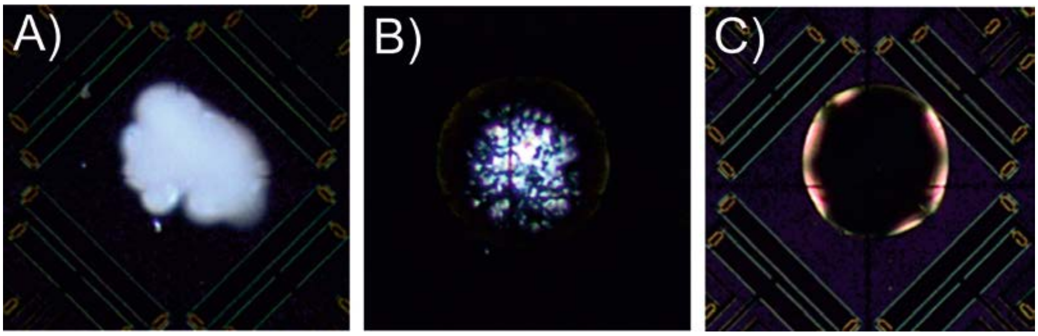


**Figure S16.** The images of microcrystalline sample **5** (*1,3-alternate*) under polarized light at heating to 95°C (A), 106°C (B) and 112°C (C).
